# Hypoxia-induced reactive oxygen species mediate N-cadherin and SERPINE1 expression, EGFR signalling and motility in MDA-MB-468 breast cancer cells

**DOI:** 10.1038/s41598-017-15474-7

**Published:** 2017-11-09

**Authors:** Iman Azimi, Rosalie M. Petersen, Erik W. Thompson, Sarah J. Roberts-Thomson, Gregory R. Monteith

**Affiliations:** 1 School of Pharmacy, The University of Queensland, Brisbane, Queensland Australia; 2 Mater Research Institute, The University of Queensland, Brisbane, Queensland Australia; 3 Translational Research Institute, Brisbane, Queensland Australia; 4 Institute of Health and Biomedical Innovation and School of Biomedical Sciences, Queensland University of Technology, Kelvin Grove, Queensland Australia; 5 University of Melbourne Department of Surgery, St. Vincent’s Hospital, Melbourne, Victoria, Australia

## Abstract

One of the hallmarks of the tumour microenvironment is hypoxia resulting from increased oxygen consumption by proliferative cancer cells and altered vasculature. Hypoxic tension initiates various cellular signals and can drive epithelial to mesenchymal transition (EMT), a process important in cancer progression. In this study, using the antioxidant N-acetylcysteine (NAC), we show that hypoxia-induced reactive oxygen species (ROS) in MDA-MB-468 breast cancer cells, selectively regulate hypoxia-induced increases in N-cadherin and SERPINE1, two proteins involved in cell adhesion. Treatment of cells with NAC also attenuated hypoxia-mediated activation of EGFR, but did not have any effect on hypoxia-mediated induction of HIF1α. Exogenous hydrogen peroxide phenocopied the effects of hypoxia on N-cadherin and SERPINE1 expression and EGFR activation, suggesting its possible involvement in these hypoxia-mediated events. Reflective of their effect on cell adhesion proteins and EGFR (associated with migratory phenotypes), NAC also reduced cell migration under hypoxic conditions, a crucial event in metastasis. Our findings suggest a selective role for redox signalling in the regulation of specific components of the responses to hypoxia and induction of EMT in breast cancer cells. This study provides new evidence supporting the potential of targeting ROS as a therapeutic strategy for the control of breast cancer metastasis.

## Introduction

Tumours rapidly exhaust the local oxygen supply creating a hypoxic environment^1^. This hypoxic microenvironment around cancer cells can promote invasion and metastasis as well as resistance to radiation therapy and anti-cancer drugs^1,2^. Cancer cells also have increased levels of reactive oxygen species (ROS) production compared to normal cells, which may contribute to tumour progression and metastasis^3–6^. ROS also play critical roles in the regulation of signal transduction pathways in a range of cellular processes and are increased by hypoxia in a number of cell types^7–9^. ROS increase in response to hypoxia occurs via the transfer of electrons from ubisemiquinone to molecular oxygen at the Q_0_ site of the mitochondrial complex III^10,11^.

Several groups have shown that hypoxia induces epithelial to mesenchymal transition (EMT) in breast cancer cells^12–15^, a process important in tumour metastasis^16^. During EMT, cancer cells acquire features of mesenchymal-like cells including enhanced migratory and invasive abilities, changes in cellular adhesion, remodelling of the extracellular matrix, and increased resistance to stress and apoptosis^17,18^. ROS can induce EMT, however, the specificity of their action in the regulation of particular signalling pathways or EMT markers is dependent on the cellular context and type of tissue and is not fully understood^19–22^. MDA-MB-468 cells are a commonly used model in the study of EMT in triple-negative breast cancer (TNBC)^23–25^, a type of breast cancer associated with high aggressiveness, poor prognosis and limited treatment options^26,27^. The EMT inducible MDA-MB-468 breast cancer cells are a PTEN mutant cell line with high levels of EGFR expression^28,29^. These features are also associated with metastasis and poor survival in TNBC patients^30,31^.

In this study we investigated the role of hypoxia-induced ROS increases in bestowing mesenchymal properties to breast cancer cells. To achieve this goal we defined the effects of ROS scavenging in the induction of EMT markers, activation of hypoxia-induced signalling pathways, and migration of breast cancer cells, and further attempted to understand the molecular mechanisms involved.

## Results

### Hypoxia increases the intracellular levels of reactive oxygen species in MDA-MB-468 cells

**Figure 1:**
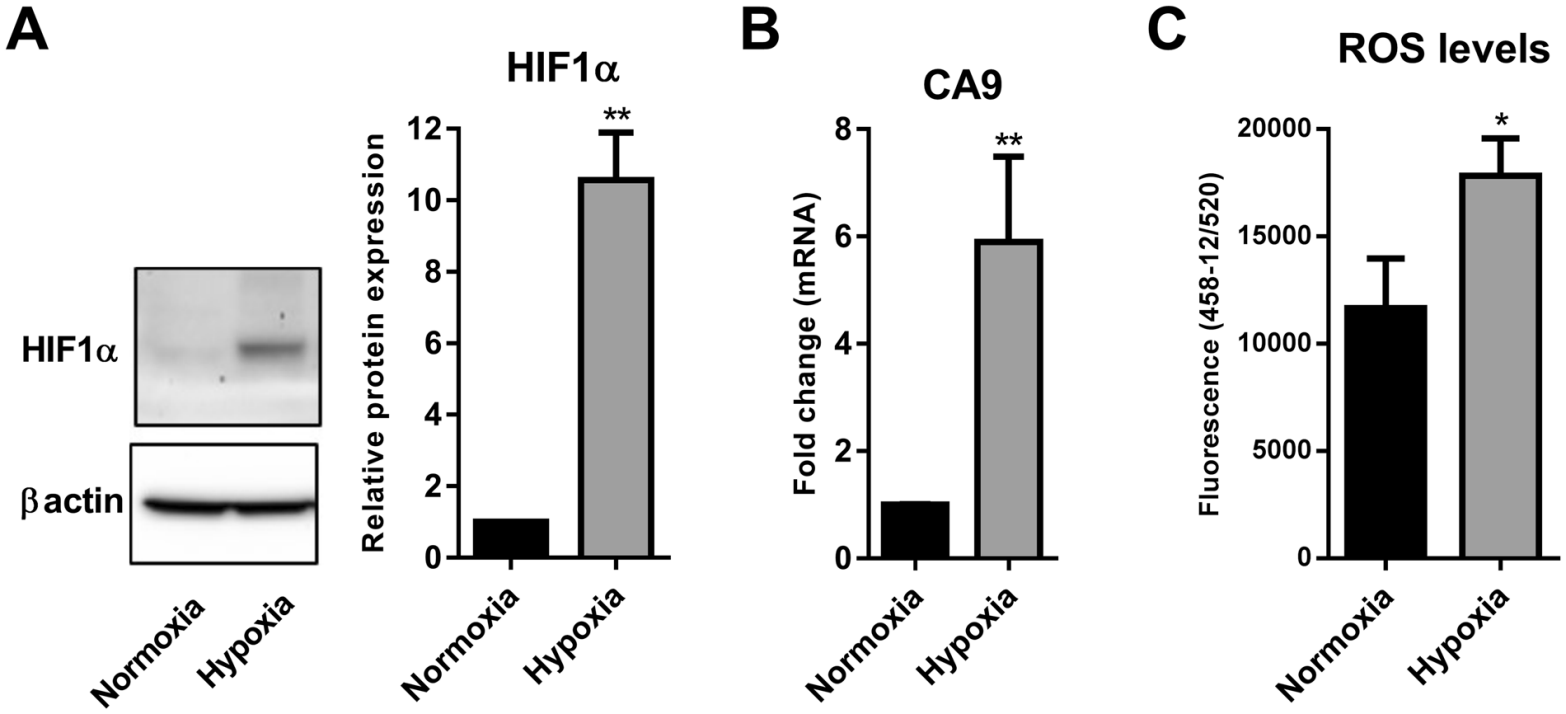
Hypoxia increases intracellular ROS levels. (**A**) Representative cropped immunoblot (left) and densitometry analysis (right) of HIF1α protein levels in MDA-MB-468 cells exposed to hypoxia (6 h) compared to normoxic cells (full-lenght immunoblot is shown in the Supplementary Fig. S2). (**B**) CA9 mRNA levels in normoxic and hypoxic (24 h) cells were assessed using real time RT-PCR. (**C**) Intracellular levels of ROS measured by DCF-DA assay in cells exposed to hypoxia (12 h) compared to control cells (remained in normoxia). Graphs represent the mean ± SD for three independent experiments. *p < 0.05, **p < 0.01 (unpaired t-test).

The induction of hypoxia (1% O_2_) in MDA-MB-468 cells was confirmed by quantifying the levels of the master regulator of hypoxia responses, hypoxia inducible factor 1- alpha (HIF1α)^32^ and an endogenous marker of hypoxic cells, carbonic anhydrase-9 (CA9)^33^. HIF-1α is stabilized via inhibition of prolyl hydroxylase domain (PHD) enzymes in the absence of oxygen, a process that can occur within a few hours of hypoxic exposure^32^. Given the rapid increase in HIF1α protein levels through hypoxia-mediated stabilization of HIF1α via inhibition of PHD enzymes^32^, and the time for gene transcription, HIF1α protein levels and target mRNA levels were assessed at 6 h and 24 h, respectively. Hypoxia significantly increased the protein levels of HIF1α (Fig. 1A) and mRNA levels of CA9 (Fig. 1B). We then assessed the intracellular levels of reactive oxygen species (ROS) using the DCF-DA assay. Exposure of MDA-MB-468 cells to hypoxia also resulted in a significant increase in intracellular ROS levels measured by DCF fluorescence (Fig. 1C). These results demonstrated the induction of hypoxic responses and the up-regulation of intracellular ROS in MDA-MB-468 breast cancer cells.

### ROS specifically controls the hypoxia-induced mRNA expression of N-cadherin and SERPINE1 but not other EMT markers

**Figure 2:**
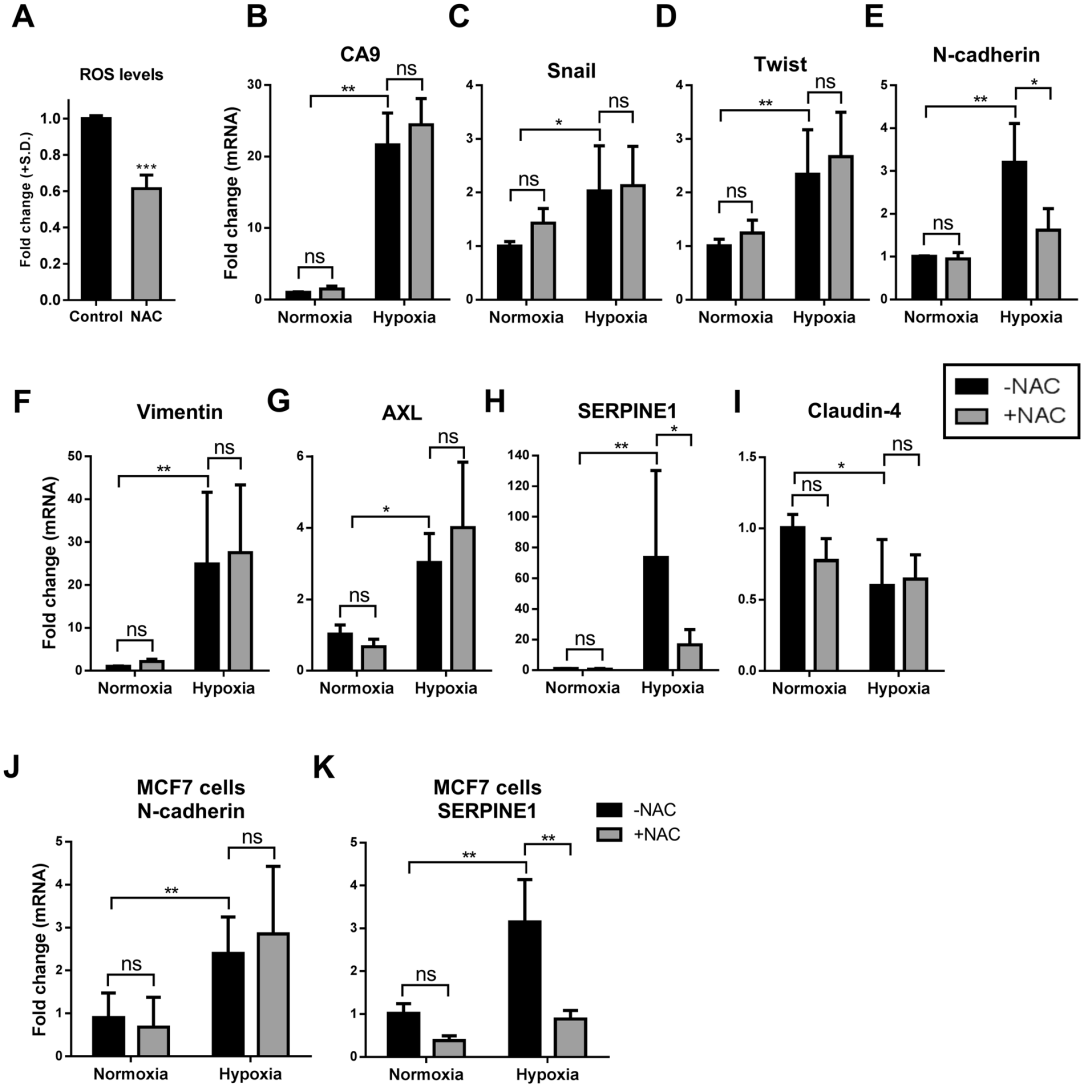
The effect of NAC treatment on mRNA levels of a panel of genes associated with EMT and hypoxic responses. (**A**) Intracellular levels of ROS measured by DCF-DA assay in cells exposed to hypoxia (12 h) in the presence and absence of NAC (10 mM). Graph represents the mean ± SD relative to the control (hypoxia with no NAC) for three independent experiments. ***p < 0.0001 (unpaired t-test). Post serum-reduction MDA-MB-468 breast cancer cells were treated with or without NAC (10 mM) and exposed to hypoxia for 24 h. The effect of NAC on (**B**) CA9, (**C**) Snail, (**D**) Twist, (**E**) N-cadherin, (**F**) vimentin, (**G**) AXL, (**H**) SERPINE1 and claudin-4 (**I**) was assessed using real time RT-PCR. ns = not significant (*p* > 0.05), *p < 0.05, **p < 0.01 (two-way ANOVA, with Tukey’s multiple comparisons test). Graphs represent the mean ± SD for three independent experiments. The effect of NAC on the expression of (**J**) N-cadherin and (**K**) SERPINE1 in hypoxia and control normoxia was assessed using real time RT-PCR. ns = not significant (*p* > 0.05), **p < 0.01 (two-way ANOVA, with Tukey’s multiple comparisons test). Graphs represent the mean ± SD for three independent experiments.

ROS can alter gene expression^34,35^. Our previous studies show a remodelling in gene transcription 24 h post hypoxia^12,36^. Given the up-regulation of ROS by hypoxia, the effect of ROS on the expression levels of some of the key genes associated with hypoxia responses was explored using the antioxidant and ROS scavenging agent NAC^37^, reducing hypoxic ROS levels by around 40% in MDA-MB-468 cells (Fig. 2A). The up-regulation of the hypoxia marker CA9 by hypoxia was not sensitive to NAC treatment (Fig. 2B). We then explored the effect of NAC on the levels of classic EMT markers, Snail, Twist, N-cadherin and vimentin (Fig. 2C–F). All of these EMT markers were significantly up-regulated by hypoxia, however, NAC treatment only attenuated hypoxia-mediated increases in N-cadherin (Fig. 2E). NAC had no effect on N-cadherin expression under normoxic conditions (Fig. 2E). The NAC sensitivity of two recently identified hypoxia-induced EMT markers (AXL and SERPINE1^24^), was then explored. AXL and SEPRPINE1 were significantly up-regulated with hypoxia by approximately 3- and 60-fold, respectively (Fig. 2G,H). NAC treatment did not have any effect on the expression levels of the AXL receptor tyrosine kinase (Fig. 2F), however, it had a dramatic inhibitory effect on hypoxia-induced increases in SERPINE1 (~75% reduction, Fig. 2H). Similar to N-cadherin mRNA regulation, NAC had no significant effect on SERPINE1 expression under normoxic conditions (Fig. 2H). Assessment of the expression of the epithelial marker Claudin-4 showed its down-regulation by hypoxia, however, this was not altered by NAC treatment (Fig. 2I). These results indicate that redox signalling is involved in the promotion of specific elements of EMT including hypoxia-induced increases in N-cadherin and SERPINE1 but not other EMT markers in MDA-MB-468 breast cancer cells. Hypoxic up-regulation of N-cadherin and SERPINE1 was not HIF1α dependent as siRNA silencing of HIF1α did not reduce this up-regulation (Supplementary Fig. S1). The effect of NAC on the hypoxia-induced expression of N-cadherin and SERPINE1 was also assessed in the luminal A breast cancer cell line MCF7. NAC had no effect on hypoxia-induced increases in N-cadherin, however, it again significantly reduced the expression of SERPINE1 induced by hypoxia in MCF7 cells (Fig. 2J and K).

### ROS mediate the activation of hypoxia-induced EGFR but not HIF1α

**Figure 3:**
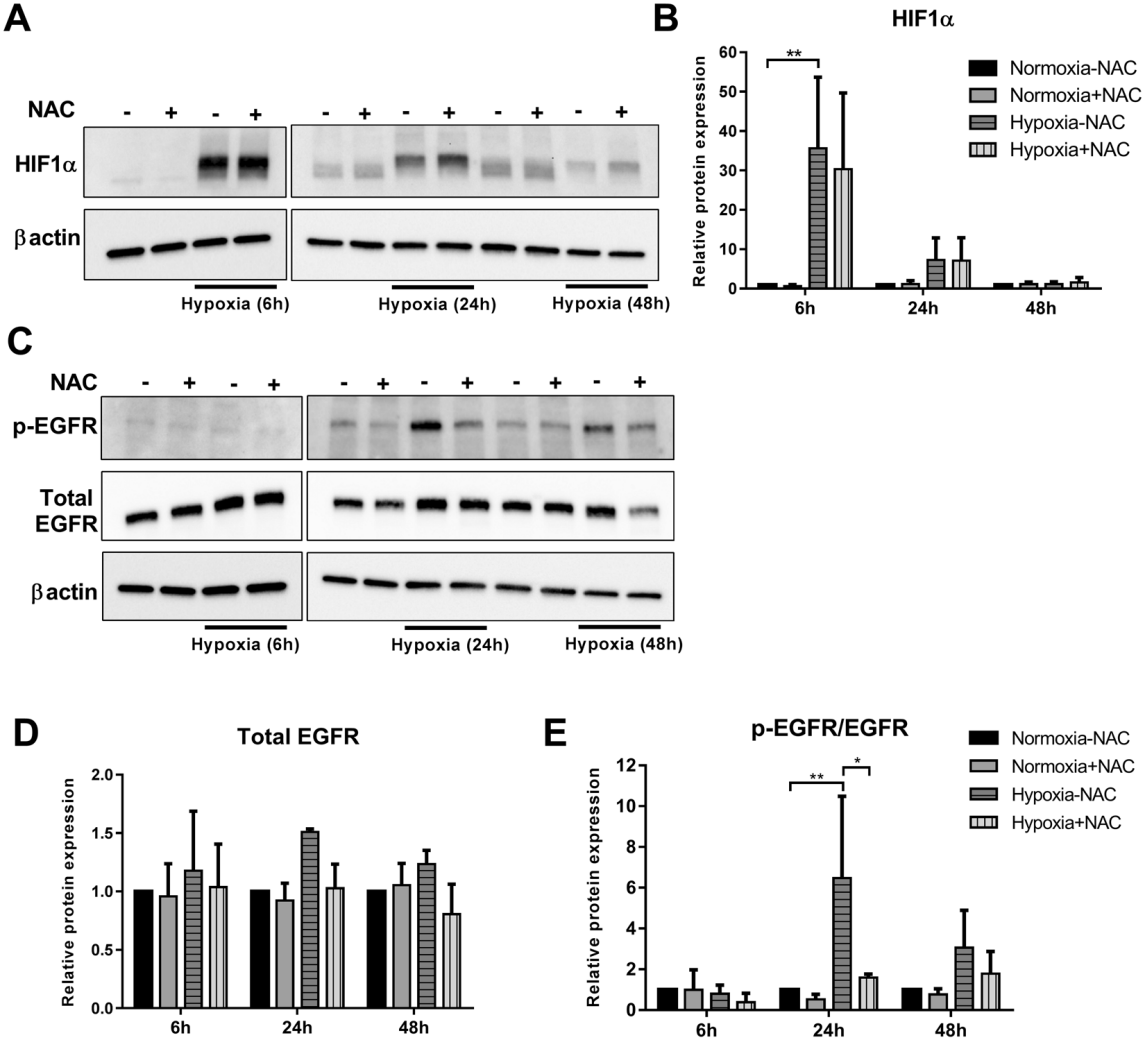
Effect of NAC on hypoxia-induced HIF1α and EGFR signalling. MDA-MB-468 cells were treated with NAC (10 mM) and exposed to hypoxia for 6 h, 24 h and 48 h. (**A**) Representative cropped immunoblot and (**B**) densitometry analysis of HIF1α protein levels (normalised to β-actin). (**C**) Representative cropped immunoblot and densitometry analysis of (**D**) total EGFR (normalised to β-actin) and phosphorylated EGFR (p-EGFR) (Y1173) levels (normalised to β-actin and total EGFR) (full-lenght immunoblots are shown in the Supplementary Fig. S2). *p < 0.01, **p < 0.001 (two-way ANOVA, with Tukey’s multiple comparisons test). All densitometry data presented are the mean of three independent experiments ± SD.

Given the redox modulation of hypoxia-induced gene expression of specific EMT markers, we sought to explore the involvement of ROS in the activation of two key hypoxia-associated signalling pathways HIF1α^32^ and EGFR^38^ at different hypoxic time points. Exposure of cells to 6 h hypoxia significantly increased the protein levels of HIF1α, however, NAC treatment did not have any effect on this induction (Fig. 3A and B). At 24 h hypoxia, there was a trend towards up-regulation of HIF1α levels with hypoxia that was also insensitive to ROS scavenging. Elevated HIF1α levels were not maintained at 48 h hypoxia (Fig. 3A and B).

Hypoxia, at any time point tested, did not significantly affect the total EGFR levels, and NAC treatment also did not change these levels (Fig. 3C and D). Maximal EGFR phosphorylation at Y1173 (the major EGFR signalling site^39^) was evident at 24 h and was significantly reduced by NAC. This increase was redox dependant as treatment with NAC significantly decreased (~70%) the hypoxia-induced EGFR phosphorylation (Fig. 3C and E). At 48 h there was a trend towards reduction of both total and activated EGFR by NAC, however, this was not statistically significant. These findings provide further evidence of the specificity in the redox modulation of cellular signals associated with hypoxia.

### Hypoxia-induced N-cadherin and SERPINE1 expression and EGFR activation are not sensitive to Trolox and Tempol

**Figure 4:**
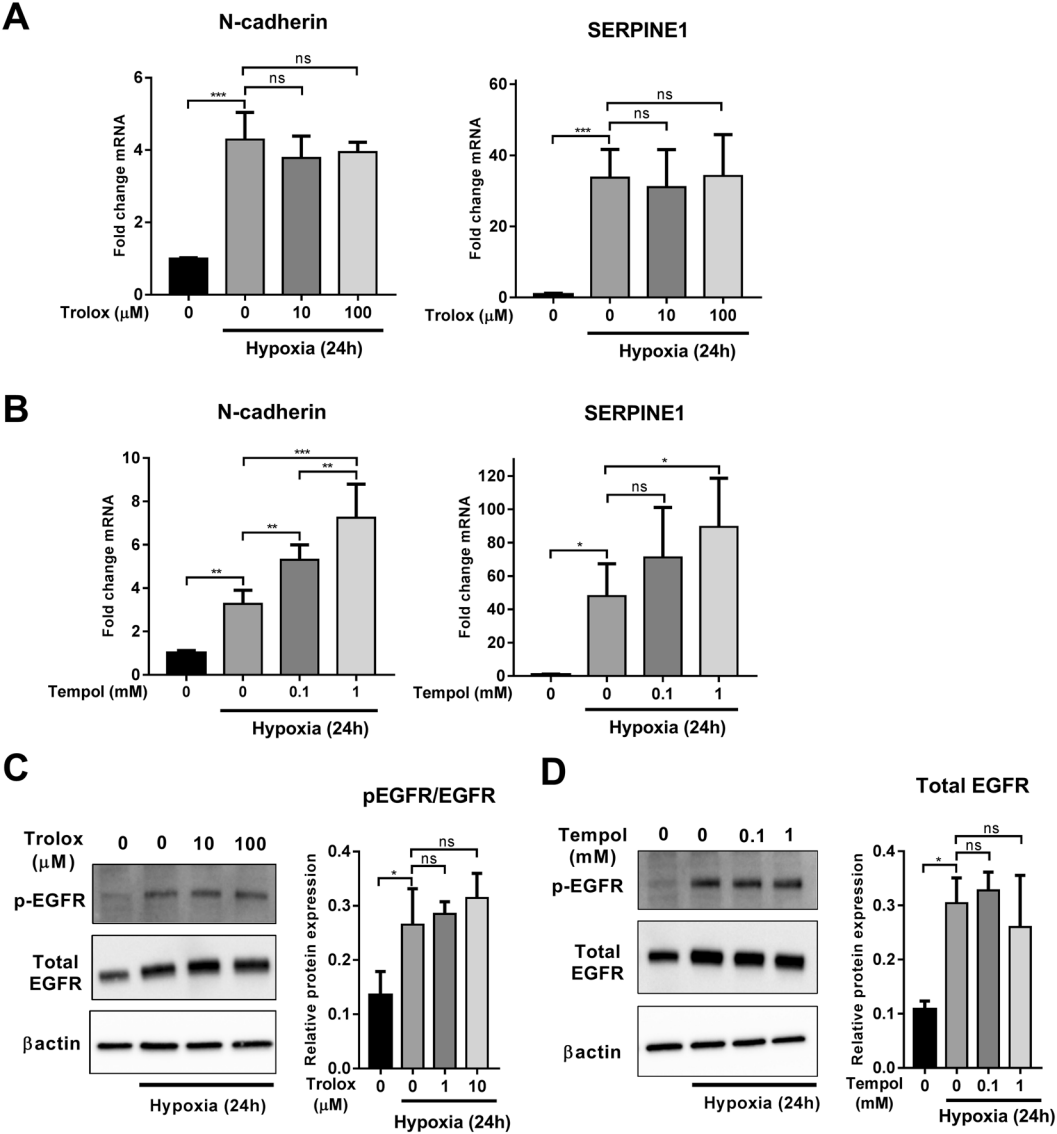
Effect of Trolox and Tempol on hypoxia-induced N-cadherin and SERPINE1 expression and EGFR activation. MDA-MB-468 cells were treated with Trolox (10 and 100 µM) and Tempol (0.1 and 1 mM) and placed under hypoxic conditions for 24 h. The mRNA levels of N-cadherin and SERPINE1 following Trolox (**A**) and Tempol (**B**) treatments were assessed using real time RT-PCR. Levels of EGFR phosphorylation (normalised to β-actin and total EGFR) following Trolox (**C**) and Tempol (**D**) treatments were assessed using immunoblotting (representative cropped immunoblots are shown in the figure; full-lenght immunoblots are shown in the Supplementary Fig. S2). ns = not significant (*p* > 0.05), *p < 0.05, **p < 0.01, ***p < 0.001 (one-way ANOVA, with Tukey’s multiple comparisons test). Graphs represent the mean ± SD for three independent experiments.

To understand the detailed mechanism of redox regulation of hypoxia-induced N-cadherin and SERPINE1 expression and EGFR activation, we treated cells with Trolox and TEMPOL, two ROS scavengers with distinct mechanisms of action. Trolox, a water-soluble vitamin E analog, inhibits lipid peroxidation by scavenging peroxyl radicals (ROO^•^)^40^. TEMPOL, on the other hand, is a superoxide dismutase (SOD) mimetic that attenuates superoxide anions^41^. Neither Trolox (10 and 100 µM) nor Tempol (0.1 and 1 mM) inhibited the induction of N-cadherin and SERPINE1 mRNA expression (Fig. 4A and B) and EGFR phosphorylation by hypoxia (Fig. 4C and D). Tempol appeared to enhance the mRNA levels of N-cadherin and SERPINE1 in a concentration dependent manner (Fig. 4B). Tempol, in addition to its superoxide anion scavenging, can also increase hydrogen peroxide (H_2_O_2_) levels^42^. This led us to investigate the effect of H_2_O_2_ on the expression of N-cadherin and SERPINE1 and phosphorylation of EGFR.

### Hydrogen peroxide induces N-cadherin and SERPINE1 mRNA expression and EGFR phosphorylation

**Figure 5:**
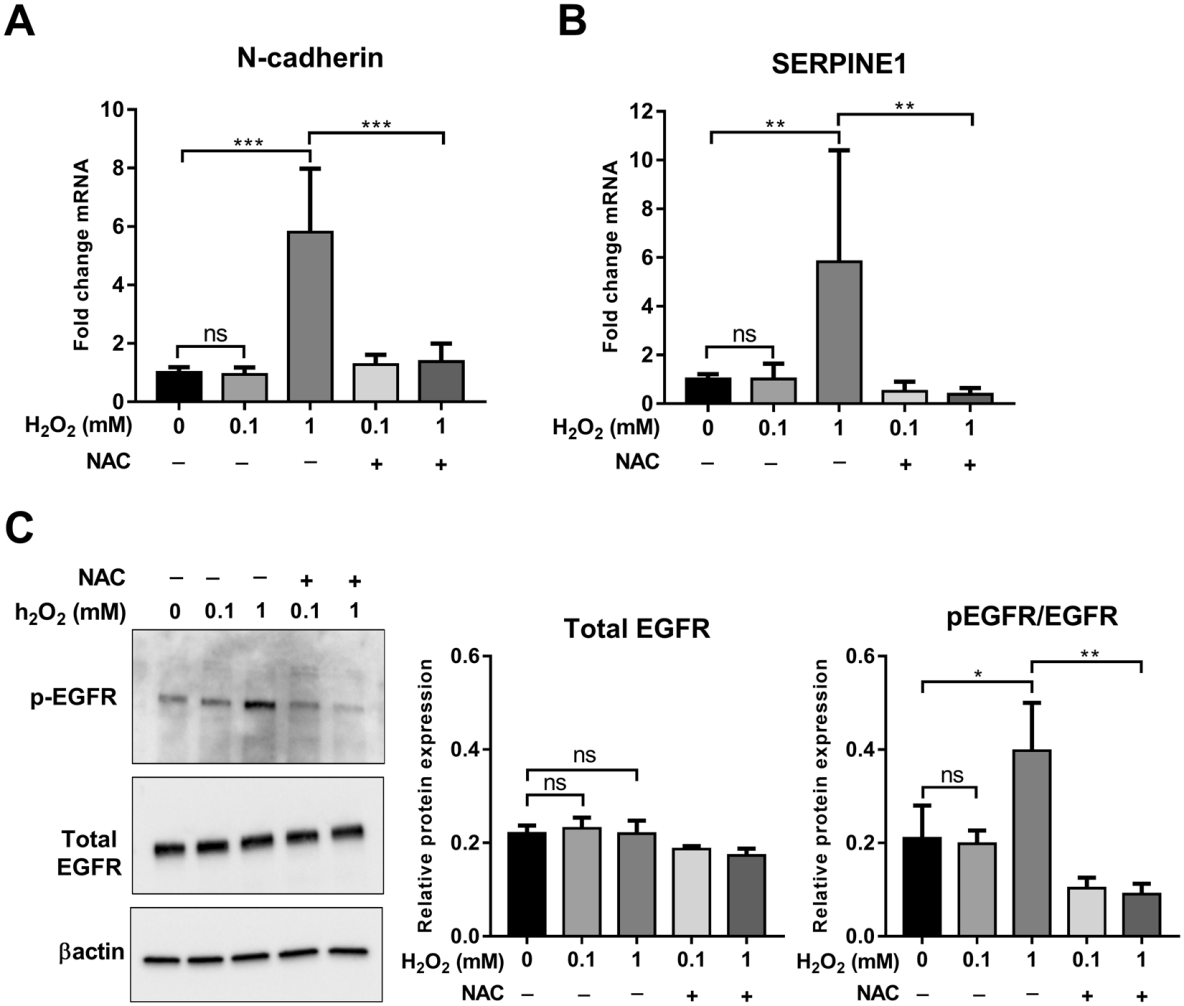
Effect of H_2_O_2_ on the expression of N-cadherin and SERPINE1 and the phosphorylation of EGFR. Post serum reduction (24 h), MDA-MB-468 cells were treated with H_2_O_2_ (0.1 and 1 mM) in the presence or absence of NAC (10 mM). The mRNA levels of N-cadherin (**A**) and SERPINE1 (**B**) were assessed using real time RT-PCR. (**C**) Representative cropped immunoblot (left) and densitometry analysis (right) of total EGFR (normalised to β-actin) and pEGFR (normalised to β-actin and total EGFR) (full-lenght immunoblots are shown in the Supplementary Fig. S2). ns = not significant (*p* > 0.05), *p < 0.05, **p < 0.01, ***p < 0.001 (one-way ANOVA, with Tukey’s multiple comparisons test). Graphs represent the mean ± SD for three independent experiments.

H_2_O_2_ is a non-radical ROS molecule^43^ that is induced by hypoxia^44,45^. We explored the effect of exogenous H_2_O_2_ treatment on the expression of N-cadherin and SERPINE1 and the activation of EGFR. Treatment of MDA-MB-468 cells with H_2_O_2_ (1 mM) in normoxic conditions resulted in a significant increase in the mRNA levels of N-cadherin and SERPINE1 (Fig. 5A and B) and EGFR phosphorylation (Fig. 5C), and this increase was attenuated by NAC. The ability of H_2_O_2_ to phenocopy the observed effects of hypoxia suggests that H_2_O_2_ may have an important role in the regulation of N-cadherin and SERPINE1 expression and EGFR activation in the hypoxia model.

### ROS contribute to the migration of MDA-MB-468 breast cancer cells under hypoxic conditions

**Figure 6:**
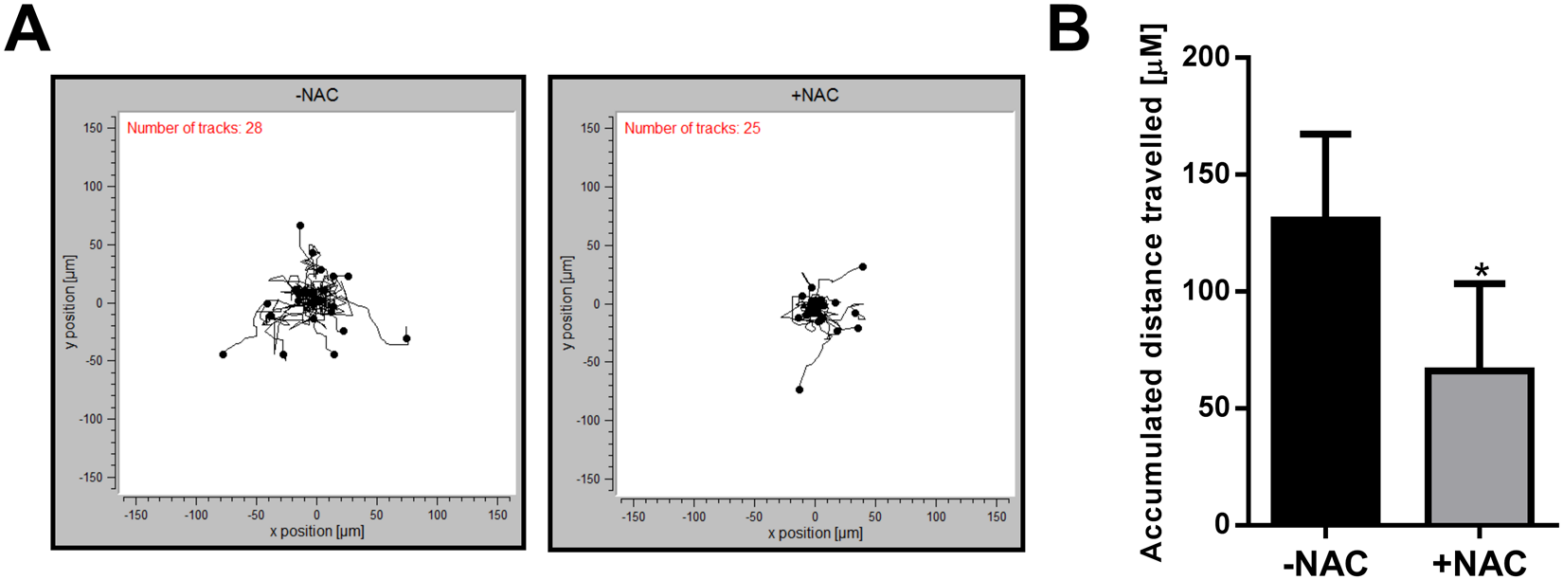
ROS scavenging with NAC reduces cell migration under hypoxic conditions. MDA-MB-468 cells were treated with NAC and exposed to hypoxia. Cell migration was analysed from 24 h to 36 h under hypoxic conditions. (**A**) Spatial plot of all cells from representative wells from the same experiment and (**B**) quantitative analysis of the mean accumulated distance travelled by cells from three independent experiment (duplicate wells) treated with NAC compared to the control cells without NAC treatment. *p < 0.05, (paired t-test).

Hypoxia can induce cell migration in a variety of cancer cell models^46–49^. Given the role of ROS in the regulation of N-cadherin and SERPINE1 (involved in matrix remodelling and cell migration^50,51^) and the effects of ROS on hypoxia-induced activation of the pro-migratory receptor EGFR^52,53^, we sought to explore the effects of NAC on the migration of MDA-MB-468 breast cancer cells during hypoxia. EMT results in a transition from collective to single cell motility and this is considered to be a crucial step in the course of tumour progression and metastasis^54,55^. Therefore, in the context of EMT, it is important to assess the migration of single cells. We used a collagen-based model and individual cell tracking system to assess and quantify the migration of MDA-MB-468 breast cancer cells. In this single-cell migration assay, the accumulative distance of individual cells migrated over a course of 12 h post exposure to hypoxia (24 h) is assessed. NAC treatment significantly decreased the migration of cells exposed to hypoxia (Fig. 6). This finding reveals the importance of ROS in bestowing cells with more migratory characteristics, which is an important consequence of EMT in breast cancer cells^18^.

## Discussion

In this study we demonstrated that hypoxia resulted in mRNA up-regulation of the hypoxic marker CA9^33^ and an up-regulation in HIF1α protein levels, as well as an increase in intracellular ROS levels in MDA-MB-468 breast cancer cells. Hypoxia also resulted in an elevation in the mesenchymal markers vimentin, N-cadherin, Snail, Twist, AXL and SERPINE1. The thiol-containing ROS scavenger NAC was used to investigate the role of redox in hypoxia-associated cellular responses. NAC is a United States Food and Drug Administration (FDA)-approved drug for the treatment of chronic obstructive lung disease (COPD)^56^ and acetaminophen (paracetamol) overdose^57^. It is also a synthetic precursor of intracellular cysteine and glutathione, which has free radical scavenging properties either directly by redox transitions of thiols, or indirectly via increasing intracellular glutathione levels^58^. NAC has anti-tumorigenic effects on tumours of the breast^59^, lung^60^ and also in Kaposi’s Sarcoma^61^. Herein we observed that ROS attenuation with NAC specifically reduced N-cadherin and SERPINE1 mRNA levels induced by hypoxia. N-cadherin and SERPINE1 are both involved in cell-cell adhesion. N-cadherin is a predominantly mesenchymal homotypic cell adhesion protein found in adherens junctions that contributes to the induction of EMT and metastasis^62^. SERPINE1, also known as plasminogen activator inhibitor type-1 (PAI-1), is an important regulatory protein involved in the extracellular matrix reorganisation and cell adhesion^63^, and is up-regulated in EMT in both EGF and hypoxia models of EMT^24^. Matrix remodelling is a crucial step for acquisition of more migratory phenotypes^64^. Assessment of the effect of NAC treatment on the expression of hypoxia-mediated N-cadherin and SERPINE1 in estrogen receptor positive (ER+), luminal-like MCF7 breast cancer cells^65^, identified a redox dependence in the regulation of SERPINE1 but not N-cadherin expression. These data suggest that hypoxia can mediate increases in N-cadherin using different molecular pathways. The potential molecular subtype specific dependence of this difference on ER expression status and/or the basal molecular phenotype should be the focus of future studies.

NAC did not show any effect on levels of classic EMT markers, Snail, Twist, AXL and vimentin suggesting the specificity of the redox signal in the regulation of distinctive aspects of EMT. This may suggest the existence of multiple regulators of the EMT phenotype. Indeed, we recently reported that the calcium permeable ion channel TRPM7 promotes epidermal growth factor (EGF)-induced EMT by selectively regulating vimentin protein expression^36,66^. Indeed, there is increasing evidence suggesting a reciprocal interplay between calcium and ROS signalling in fine tuning cellular signalling networks^67^.

We also investigated the effect of ROS scavenging with NAC on the levels of HIF1α protein and EGFR phosphorylation, two of the key hypoxia-associated signalling pathways^32,38^. NAC had no significant effect on the protein levels of the hypoxia master regulator, HIF1α, under normoxic or hypoxic conditions. In contrast, in human hepatoma 3B, fibrosarcoma, and epithelial lung cells HIF1α stabilisation during hypoxia is redox sensitive^11,68,69^. However, studies by Chua *et al*. in human osteosarcoma found that hypoxic HIF1α stabilisation occurs independently of mitochondrial ROS^70^ such as we observed in our model. Clearly the cellular context and tissue specific role of ROS in responses to hypoxia is important. Our studies did identify a critical role for ROS with hypoxia-mediated activation of EGFR. EGFR is overexpressed in TNBC, where its inhibition can induce a change from a mesenchymal to a more epithelial phenotype^71^. The two tested ROS scavengers, Trolox (scavenger of peroxyl radicals^40^) and Tempol (scavenger of superoxide anions^41^), did not attenuate the hypoxia-induced expression of N-cadherin and SEPRINE1 and phosphorylation of EGFR at the tested concentrations, suggesting the non-involvement of these free radicals in the observed hypoxic events. Instead, N-cadherin and SEPRINE1 mRNA levels were significantly up-regulated by increasing concentrations of Tempol. This effect could have been due to elevated H_2_O_2_ levels since induction of H_2_O_2_ is a property of Tempol in addition to its role as a superoxide scavenger^42^. Indeed, treatment of cells with H_2_O_2_ in normoxia phenocopied the effect of hypoxia on the up-regulation of N-cadherin and SEPRINE1 expression and EGFR phosphorylation, suggesting the involvement of H_2_O_2_ in these hypoxia-mediated events. H_2_O_2_ is a stable diffusable non-radical oxidant and is involved in many biological processes such as signal transduction, cell differentiation and proliferation^72^. In the context of our studies (showing H_2_O_2_ induction of SERPINE1 and N-cadherin mRNA and EGFR phosphorylation), it is interesting to note that H_2_O_2_ can also regulate the activity of transcription factors^73^ and protein phosphorylation via cysteine oxidation^74^ in other models. Future studies could assess the role of potential transcription factors in the up-regulation of ROS-mediated SERPINE1 and N-cadherin. Given our findings of a link between redox signalling and the induction of N-cadherin and SERPINE1 and EGFR activation, and since each of these proteins are involved in the promotion of cell migration^50,51,75^, we investigated the effect of ROS scavenging on the migration of MDA-MB-468 cells. A collagen-based cell migration model revealed that cells treated with NAC showed significantly less motility under hypoxic conditions. Transition from collective to single cell migration is a consequence of EMT, and is an important step towards tumour invasion and metastasis^54,55^. Metastasis is responsible for about 90% of human cancer death, and is influenced by the tumour microenvironment^76^, which is often low in O_2_ and high in pro-oxidants^77^. Our studies provide further evidence for a role of ROS in contributing to breast cancer progression particularly in the context of the hypoxic tumour microenvironment. This work also shows that there are specific events induced by hypoxia that occur independently of ROS, including specific EMT processes associated with vimentin, Snail and Twist induction. Since these events may be critical to aspects of disease progression including therapeutic resistance^78^ any therapeutic targeting of metastatic progression associated with hypoxia will require an approach encompassing the targeting of redox-dependent and redox-independent processes. Indeed, our results may help explain why the protective effects against metastasis of ROS scavengers has shown varied success *in vivo* and in clinical studies^79,80^.

In conclusion, in this study we have demonstrated a role for redox signalling in the promotion of hypoxia-induced changes in specific EMT markers and in the regulation of specific hypoxic cellular responses. We have also defined a critical role for ROS in MDA-MB-468 breast cancer cell migration under hypoxic conditions. Further studies are warranted to explore the mechanism of the regulation of specific hypoxia-associated cellular responses by ROS.

## Materials and Methods

### Cell culture

MDA-MB-468 and MCF7 breast cancer cells^12^ were cultured in Dulbecco’s Modified Eagle’s Medium (DMEM; D6546 Sigma- Aldrich, St Louis, MO, USA) supplemented with 10% foetal bovine serum (FBS) and 4 mM L-glutamine and maintained in a humidified incubator (37 °C with 5% CO_2_). For the induction of hypoxia, 24 h post seeding, cells were serum reduced (0.5% FBS, 24 h) and placed in a Sanyo MCO-18M multi-gas incubator with 1% O_2_ for the indicated time points (see figure legends). Time points were selected based on preliminary data and literature reports of hypoxia mediated changes in this and/or a similar model^12,36,81^. Control cells (normoxia) were kept in the humidified incubator with 21% O_2_ for the matching time points. MDA-MB-468 and MCF7 cells were cultured for less than 10 passages (5–6 weeks) and were regularly screened for mycoplasma contamination (MycoAlert; Lonza Basel, Switzerland). Cell line STR profiling was confirmed by QIMR Berghofer Medical Research Institute, Brisbane, Australia using the StemElite ID Profiling Kit (Promega, Madison, WI, USA).

### Antioxidants and hydrogen peroxide treatments

The three antioxidants used in this study were purchased from Sigma-Aldrich and were as follow: N-Acetyl-L-cysteine (NAC) (Cat# A7250), Trolox (( ± )-6-Hydroxy-2,5,7,8-tetramethylchromane-2-carboxylic acid) (Cat# 238813) and Tempol (4-Hydroxy-2,2,6,6-tetramethylpiperidine 1-oxyl) (Cat# 176141). Hydrogen peroxide (H_2_O_2_) was purchased from Cell Biolabs as one of the components of the OxiSelect^TM^ Intracellular ROS Assay Kit (STA-342). All the antioxidants and H_2_O_2_ were added to MDA-MB-468 cells in serum reduced-media (0.5% FBS) and incubated with cells for 24 h prior to RNA or protein isolation.

### Real time RT-PCR

Total RNA from cells was isolated and purified using the RNeasy Plus Mini Kit (Qiagen, Hilden, Germany). Omniscript RT Kit (205111, Qiagen) was used for the reverse transcription of the purified RNA. The target cDNA was amplified under universal cycling conditions in a StepOnePlus^TM^ instrument (Applied Biosystems) using TaqMan® gene expression assays and the TaqMan® Fast Universal PCR Master Mix (Applied Biosystems, Carlsbad, CA, USA). The relative target quantity was determined using the comparative C_T_ (ΔΔC_T_) method and normalised to 18 S ribosomal RNA.

### Immunoblotting

Protein samples were resolved on NuPAGE Novex 4–12% Bis-Tris Gradient Gel (Invitrogen, Waltham, MA, USA) by SDS gel electrophoresis and transferred to polyvinylidene difluoride membrane (PVDF). Primary antibodies used in this study were as follows: 1:1000 dilution of EGFR (2232) and phospho-EGFR (Tyr 1173) (4407) antibodies from Cell Signalling Technology (Danvers, MA, USA), 1:500 dilution of HIF1α antibody from BD Bioscience (Bedford, MA, USA; 610959), 1:10,000 dilution of β-actin antibody from Sigma-Aldrich (A5441). Horseradish peroxidase-conjugated goat anti-mouse (170–6516) and goat anti-rabbit (170–6515) secondary antibodies were purchased from Bio-Rad (Hercules, CA, USA) and used at a 1:10,000 dilution. Primary antibodies were incubated with PVDF membranes overnight at 4 °C (with the exception of β-actin antibody - 1 h at room temperature). Secondary antibodies were incubated for 1 h at room temperature. Protein bands were detected with chemiluminescence using SuperSignal^TM^ West Dura Substrate (34075, Thermo Fisher Scientific, Waltham, MA, USA) and visualised using a Bio-Rad VersaDoc Imaging System or a Bio-Rad ChemiDoc Imaging System. The intensity of bands was quantified with Image Lab V 5.2.1 software (Bio-Rad) and normalised to β-actin controls. Phosphorylated proteins were then normalised to their respective total protein level.

### Measurement of intracellular ROS

Intracellular ROS levels was measured using the OxiSelect^TM^ Intracellular ROS Assay Kit (STA-342, Cell Biolabs, San Diego, CA, USA), which uses a cell-permeable fluorogenic 2′, 7′-dichlorodihydrofluorescin diacetate (DCFH-DA) that after being diffused into cells is deacetylated by cellular esterases to non-fluorescent 2′, 7′-dichlorodihydrofluorescin (DCFH), which is oxidised to highly fluorescent 2′, 7′-dichlorodihydrofluorescin (DCF) by ROS. The fluorescent intensity is proportional to the intracellular ROS levels within the cytosol^82^. Cells were seeded at 2 × 10^5^ per well in 96-well plates. Post seeding (24 h), cells were serum reduced for a further 24 h and then placed in a hypoxic incubator (or remained in the normoxic incubator for the control group) for 12 h. For the measurement of intracellular ROS, cells were gently washed twice with phosphate-buffered saline (PBS) and then incubated with 10 µM DCFH-DA for 30 min at 37 °C. The fluorescent intensity was measured using a FLUOstar Omega Microplate Reader (BMG LABTECH, Ortenberg, Germany) with excitation at 485–512 nm and emission at 520 nm. Fluorescence background was subtracted from all samples.

### Cell migration assay

Collagen matrices were prepared for the assessment of cell motility. Cell culture plates (96-well) were coated with 50 µL of a collagen mixture containing 10X PBS (8% v/v), DMEM (24% v/v), collagen type I from bovine skin (final concentration 2 mg/mL; C4243, Sigma-Aldrich), brought to physiological pH with 1 M NaOH. Collagen gels were formed by incubating plates at 37 °C, 5% CO_2_ for 1 h. Cells were then seeded at a very low density (to ensure the assessment of single cell movement) of 500 cells per well on top of the collagen gel. After serum reduction (0.5% FBS) for 24 h, cells were treated with NAC (10 mM) and placed on the stage of a JuLi^TM^ Stage Live Cell Imaging System (NanoEnTek Inc. Seoul, South Korea) housed in a hypoxia incubator. Bright field images from the centre of wells were taken every 15 min using a 4X objective. Assessment of cell motility was performed 24 h post exposure to hypoxia for a period of 12 h (between 24 h and 36 h in hypoxia). Strict criteria were implemented in the exclusion of cells for quantification of their motility. Exclusion criteria included the removal of cells that died, divided or moved in or out of the field of view during the time of assessment. After implementing these exclusion criteria, all cells within the field of view (ranging from 17 to 48 cells), were selected for assessment of their migration during the 12 h period. Cells were individually tracked using the Manual Tracking plug-in of ImageJ 1.49q software (NIH, Bethesda, MD, https://imagej.nih.gov/ij/). The position of cells in each frame taken every 15 min was recorded. Chemotaxis and Migration Tool V2.0 (Ibidi, Munich, Germany) was used for the illustration of cell movements and calculation of accumulated distance travelled by each cell.

### Statistical analysis

GraphPad Prism version 6.05 for Windows (GraphPad Software Inc, La Jolla, CA, USA) was used for statistical analysis. Statistical tests used are described in each figure legend. Experiments were performed in triplicate and data are presented as mean ± SD.

## Electronic supplementary material

Supplementary information
