# Supplementary material for: Hypoxia-induced reactive oxygen species mediate N-cadherin and SERPINE1 expression, EGFR signalling and motility in MDA-MB-468 breast cancer cells
Source: Sci Rep. 2017 Nov 9;7:15140. doi: 10.1038/s41598-017-15474-7 (PMC5680275; doi:10.1038/s41598-017-15474-7)
Supplement: Supplementary file 1 — Supplementary information [file 41598_2017_15474_MOESM1_ESM.pdf]

**Hypoxia-induced reactive oxygen species mediate N-cadherin and SERPINE1 expression, EGFR signalling and motility in MDA-MB-468 breast cancer cells**

Iman Azimi[1, 2, 3, *], Rosalie M. Petersen[1], Erik W. Thompson[3,4,5], Sarah J. Roberts-Thomson[1], Gregory R. Monteith[1,2,3]

[1]School of Pharmacy, The University of Queensland, Brisbane, Queensland, Australia

[2]Mater Research Institute, The University of Queensland, Brisbane, Queensland, Australia

[3]Translational Research Institute, Brisbane, Queensland, Australia

[4]Institute of Health and Biomedical Innovation and School of Biomedical Sciences, Queensland University of Technology, Kelvin Grove, Queensland, Australia

[5]University of Melbourne Department of Surgery, St. Vincent's Hospital, Melbourne, Victoria, Australia

* Corresponding author: Iman Azimi, Email address: i.azimi@uq.edu.au

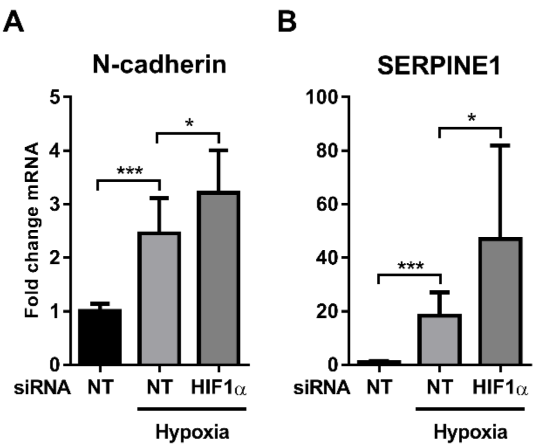


**Supplementary Figure S1**. The effect of HIF1α silencing on the expression of N-cadherin and SERPINE1. The effect of siRNA-mediated HIF1α silencing relative to non-targeting control (NT) on hypoxia-induced N-cadherin (A) and SERPINE1 (B) mRNA up-regulation (24 h hypoxia). * $p < 0.05$, ** $p < 0.001$ (one-way ANOVA, with Tukey's multiple comparisons test), n = 3 ± SD.

# Fig. 1A

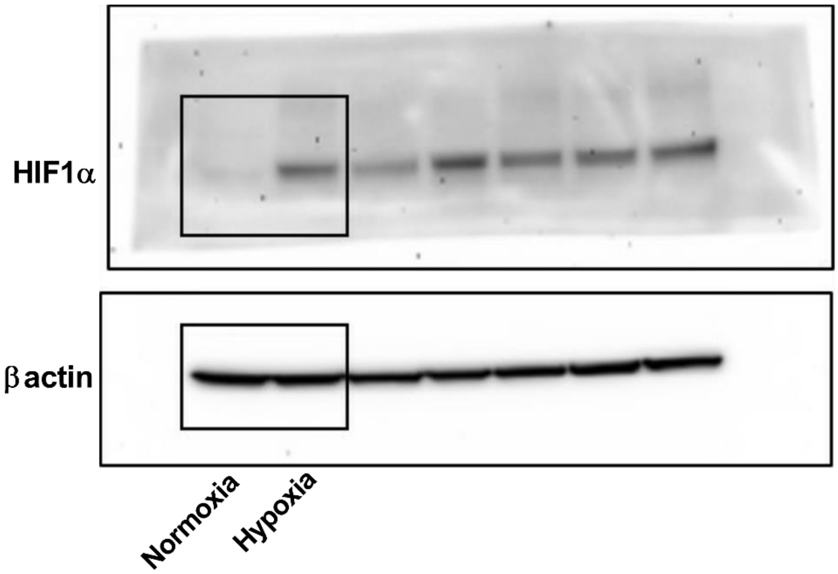


# Fig. 3A

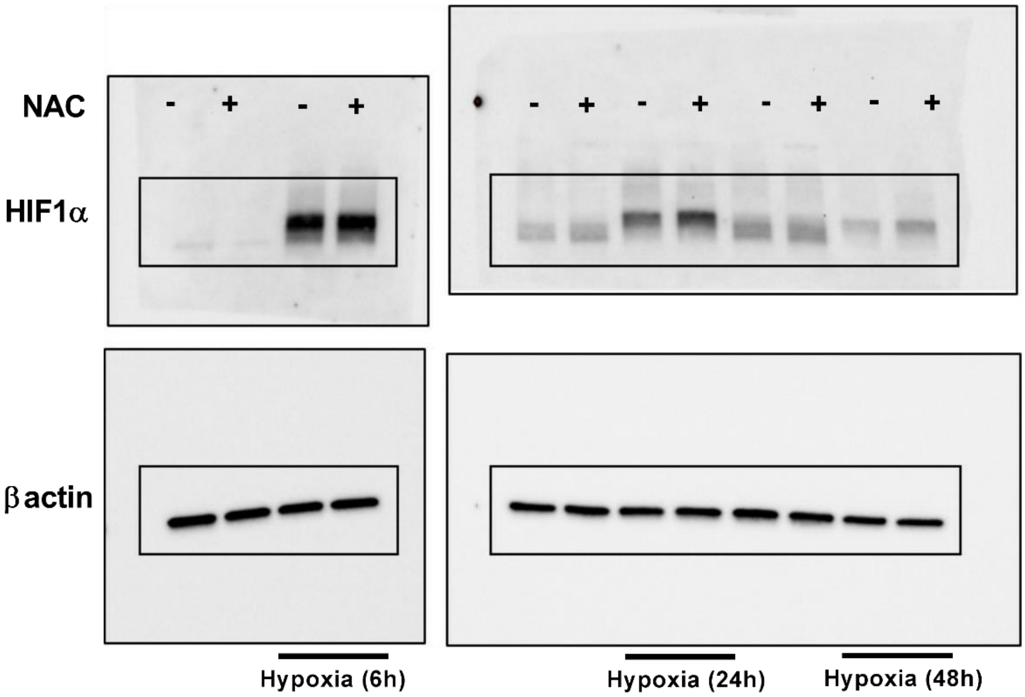


**Supplementary figure S2**. The full-length uncropped images of all immunoblots with indicated areas of selection highlighted in black.

## Fig. 3C

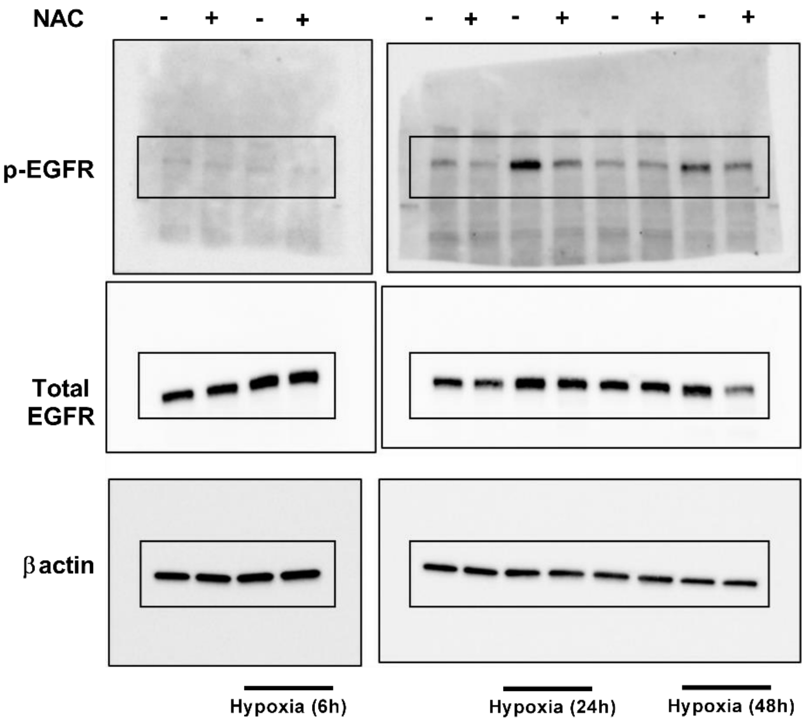


## Fig. 4C

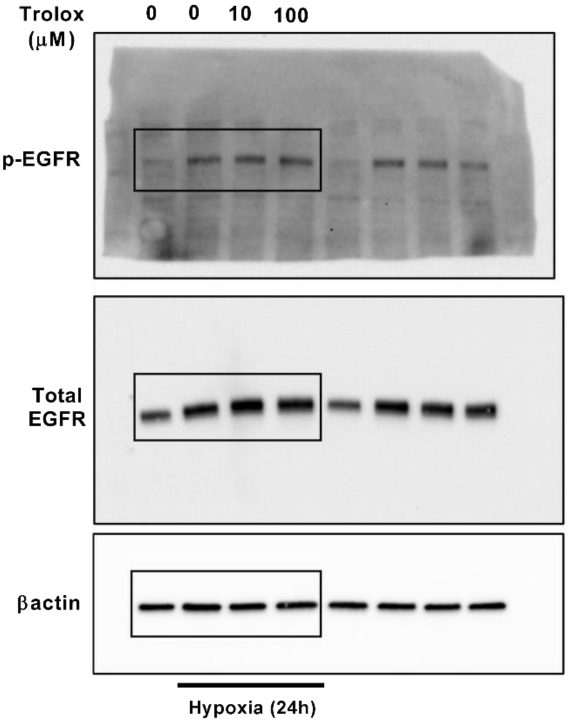


**Supplementary figure S2**. The full-length uncropped images of all immunoblots with indicated areas of selection highlighted in black.

Fig. 4D

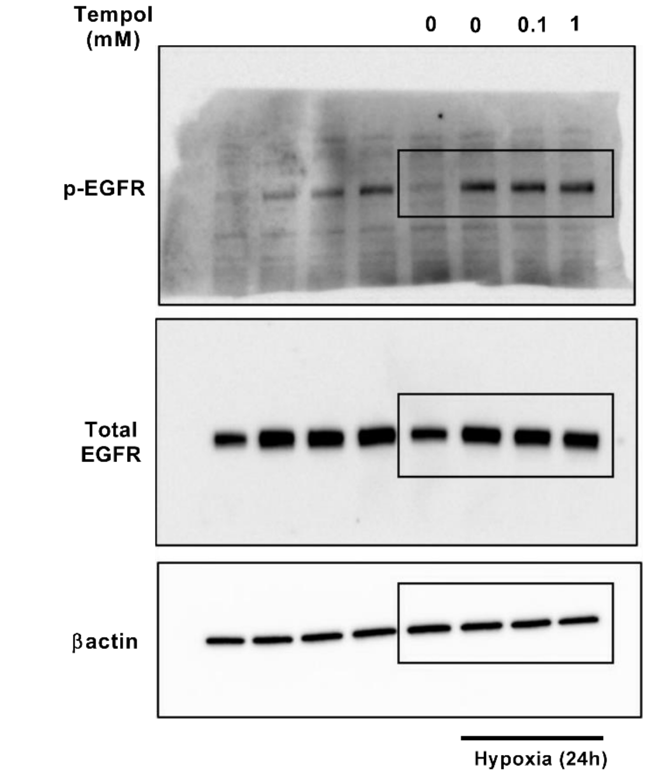


Fig. 5C

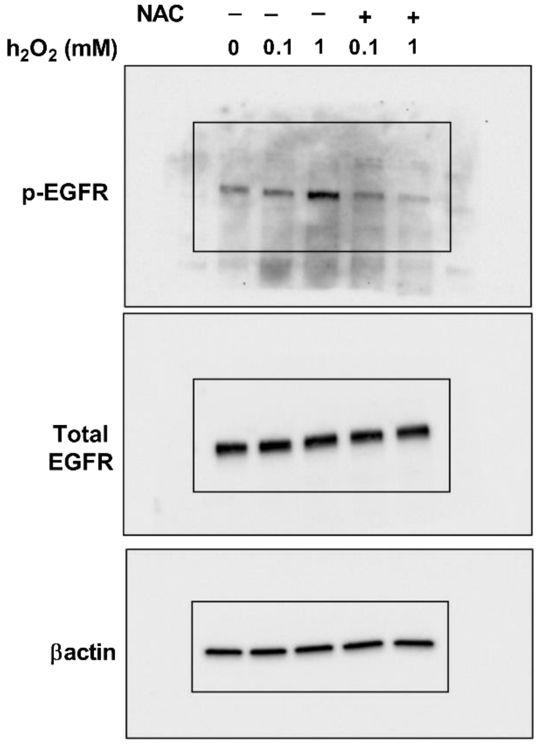


**Supplementary figure S2**. The full-length uncropped images of all immunoblots with indicated areas of selection highlighted in black.
